# Use of health services and perceived need for information and follow-up after percutaneous coronary intervention

**DOI:** 10.1186/s13104-023-06662-y

**Published:** 2024-01-05

**Authors:** Nina Hjertvikrem, Gunhild Brørs, Irene Instenes, Charlotte Helmark, Trond Røed Pettersen, Svein Rotevatn, Ann Dorthe O. Zwisler, Tone Merete Norekvål, the CONCARDPCI Investigators

**Affiliations:** 1 Department of Heart Disease, Haukeland University Hospital, Bergen, Norway; 2 Clinic of Cardiology, St. Olavs University Hospital, Trondheim, Norway; 3 Faculty of Health and Caring Sciences, Western Norway University of Applied Sciences, Bergen, Norway; 4 Department of Cardiology, Zealand University Hospital, Roskilde, Denmark; 5 Department of Cardiology, Odense University Hospital, Odense, Denmark; 6 REHPA, Knowledge Centre for Rehabilitation and Palliative Care, Nyborg, Denmark; 7 Department of Clinical Science, University of Bergen, Bergen, Norway

**Keywords:** Follow up care, Health services, Percutaneous coronary intervention

## Abstract

**Objective:**

Few patients achieve full control of their coronary artery disease (CAD) risk factors. Follow-up, such as cardiac rehabilitation, is important to increase adherence to lifestyle changes and treatment, to improve the patient’s risk profile, and to treat established complications of CAD clinical events. However, the type of follow-up patients receive varies. Therefore, the aim of this research note was to describe and compare patients’ self-reported use of health services, the type of follow-up patients reported to prefer, and the type of information patients reported to be important, in two countries with different follow-up practices after PCI.

**Results:**

We included 3417 patients in Norway and Denmark, countries with different follow-up strategies after PCI. The results showed large differences between the countries regarding health services used. In Denmark the most frequently used health services were consultations at outpatient clinics followed by visits to the general practitioner and visits to the fitness centre, whereas in Norway visits to the general practitioner were most common, followed by rehospitalisation and no follow-up used. However, patients found the same type of follow-up and information important in both countries. Patients’ perceived need for follow-up and information decreased over time, suggesting a need for early follow-up when the patients are motivated.

*Trial registration*: NCT03810612 (18/01/2019).

**Supplementary Information:**

The online version contains supplementary material available at 10.1186/s13104-023-06662-y.

## Introduction

After percutaneous coronary intervention (PCI), the European Society of Cardiology (ESC) guidelines [1, 2] recommend initiating secondary preventive strategies, as well as enabling patients to self-manage their own lifestyle. Secondary prevention and cardiac rehabilitation (CR) for patients with coronary artery disease (CAD) consist of an extensive set of treatment options ranging from optimal medical therapy, lifestyle interventions and stress management [1, 3]. Despite proven benefits on both morbidity and mortality [4, 5], few patients achieve complete risk factor control [6]. The quality of the secondary prevention offered to patients varies between and within countries [7–9]. Furthermore, CR participation rates are reported to be less than 50% [10–12]. Geographical accessibility and lack of continuity in the healthcare system have been identified as barriers to participation [13]. In Denmark, patients are routinely referred to secondary prevention and CR [14], while Norway does not practice routine referral to any kind of follow-up. For many patients, the general practitioner (GP) is therefore a key person to initiate and coordinate secondary prevention strategies and provide long-term follow-up.

Understanding patients’ own preferences as regards the provision of secondary prevention and CR after PCI can potentially increase adherence to treatment and lifestyle changes [15, 16]. Therefore, the aim of this research note was to describe and compare patients’ self-reported use of health services, the type of follow-up patients reported to prefer, and the type of information patients reported to be important, in two countries with different follow-up practices after PCI.

## Main text

### Methods

Real-world data from 3417 patients at three Norwegian and four Danish referral PCI centres were collected in the prospective multicentre CONCARD^PCI^ cohort study between June 2017 and May 2020 [17]. Patients were eligible to participate if they gave informed consent, were undergoing PCI with stent implantation according to diagnostic criteria set out in ESC guidelines [18], were ≥ 18 years of age, and living at home at the time of inclusion. Those who did not speak Norwegian/Danish or were unable to complete the questionnaires due to reduced capacity, or who were institutionalised, had a life expectancy of less than one year, or had undergone PCI without stent implantation, in connection with transcatheter aortic valve implantation or MitraClip examination, or who had previously been enrolled in CONCARD^PCI^ (readmissions) were excluded (see Additional file 1). Comparison of participants and those declining participation in the study for the Norwegian centres is available in Additional file 2.

De-novo-created questions were developed by the CONCARD^PCI^ investigators to assess patients’ use of health services, the type of follow-up patients prefer, and information patients find important at 2-, 6- and 12-month follow-up. Development of the questions were based on in-depth interviews performed prior to this study. The three de novo-questions reported here were: (1) During the last two months, which of the following health services have you used? (Multiple answers possible from predefined answers ranging from general practitioner to alternative treatment, including none of the above). (2) Looking back at the last two months, which type of follow-up would you have preferred? (Multiple answers possible from predefined answers ranging from cardiac rehabilitation daytime 3–5 weeks, to internet-based follow-up, including do not want follow-up). (3) Looking back at the last two months, what type of information is important in the follow-up? (Multiple answers possible from predefined answers ranging from information about heart medication to sexuality, including do not need information). At 6- and 12-month follow-up the questions were phrased in the last six months.

## Results

Patients were predominantly male (78%), with a mean age of 66 years (SD 11, range 20–96 years), and cohabitating (76%). Most patients had a lower educational level (20% primary school and 43% vocational school). Acute coronary syndrome was the most frequent cause of admission for PCI (62%), and 26% had previously undergone PCI. Patient characteristics are presented in Additional file 3.

**Fig. 1:**
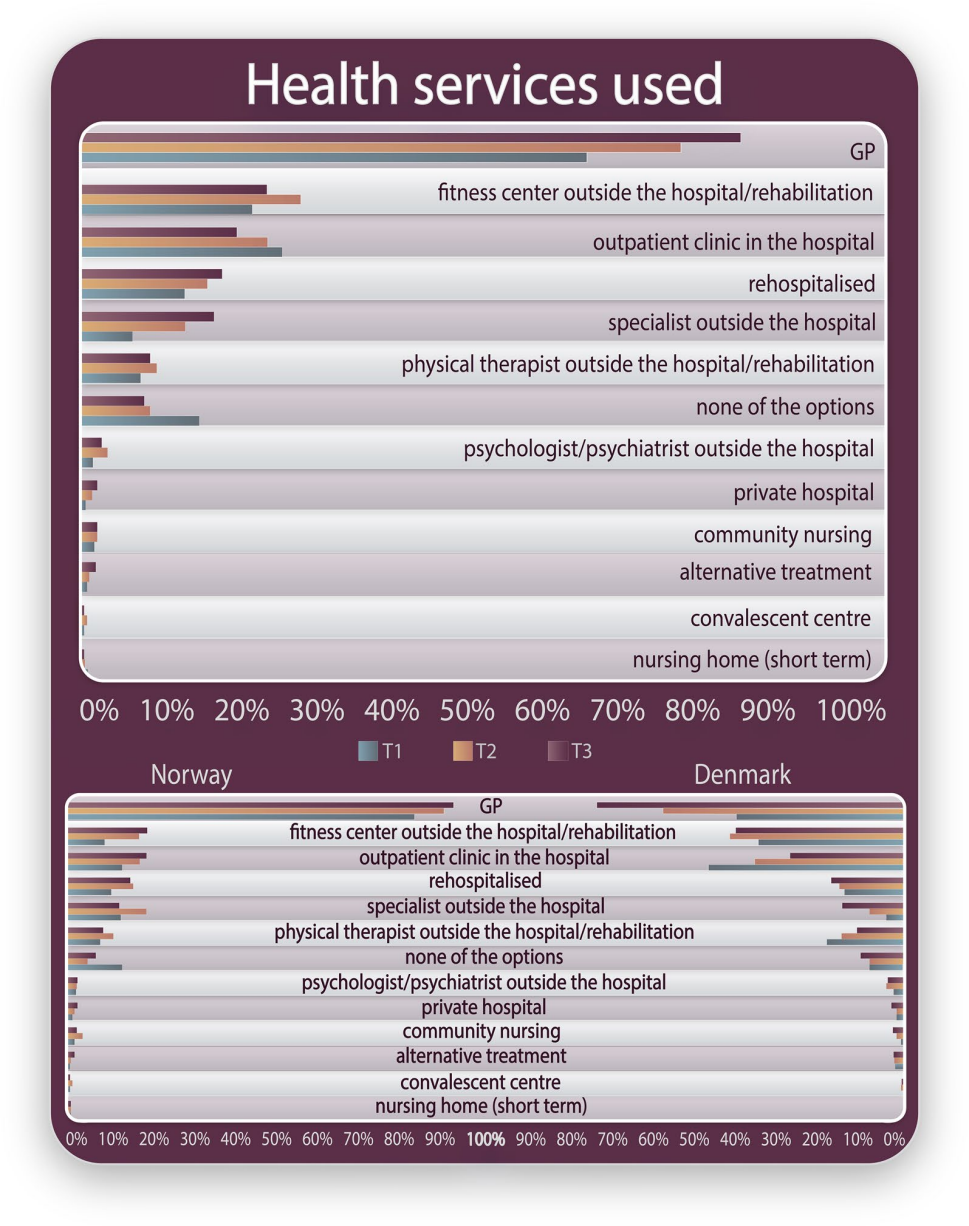
Patient reported use of health services after percutaneous coronary intervention

Figure 1 shows the type of health services patients reported to have used after PCI, (see Additional file 4 for Health services used in a table version). In Norway, more than 80% reported visiting their *GP* at 2-month follow-up, compared to 40% in Denmark. Moreover, consultations at an *outpatient clinic* were more common in Denmark compared to Norway at 2-month follow-up (46% vs. 10%). Following *GP* and *rehospitalisation* (13%), *not having had any follow-up* (13%) was most reported by the Norwegian patients. For the Danish patients, consultations at an *outpatient clinic* were followed by visits to the *GP* (40%) and going to the *fitness centre* (34%) as the most used services. The proportion of patients not participating in any follow-up care was higher in Denmark (18%) than Norway (13%). The rehospitalisation rate for Danish patients (14%) was similar to the Norwegian patients (13%). For the Norwegian patients, 10% had attended an outpatient clinic and 12% a fitness centre. In Denmark, the same top 3 health services were used at 12-month follow-up, although the order was different. In Norway, the *GP* was most used (92%), *specialist outside hospital* second (19%) and *rehospitalised* third (19%).

At 12-month follow-up, 11% of Danish patients and 6% of Norwegian patients reported not having used any health services.

**Fig. 2:**
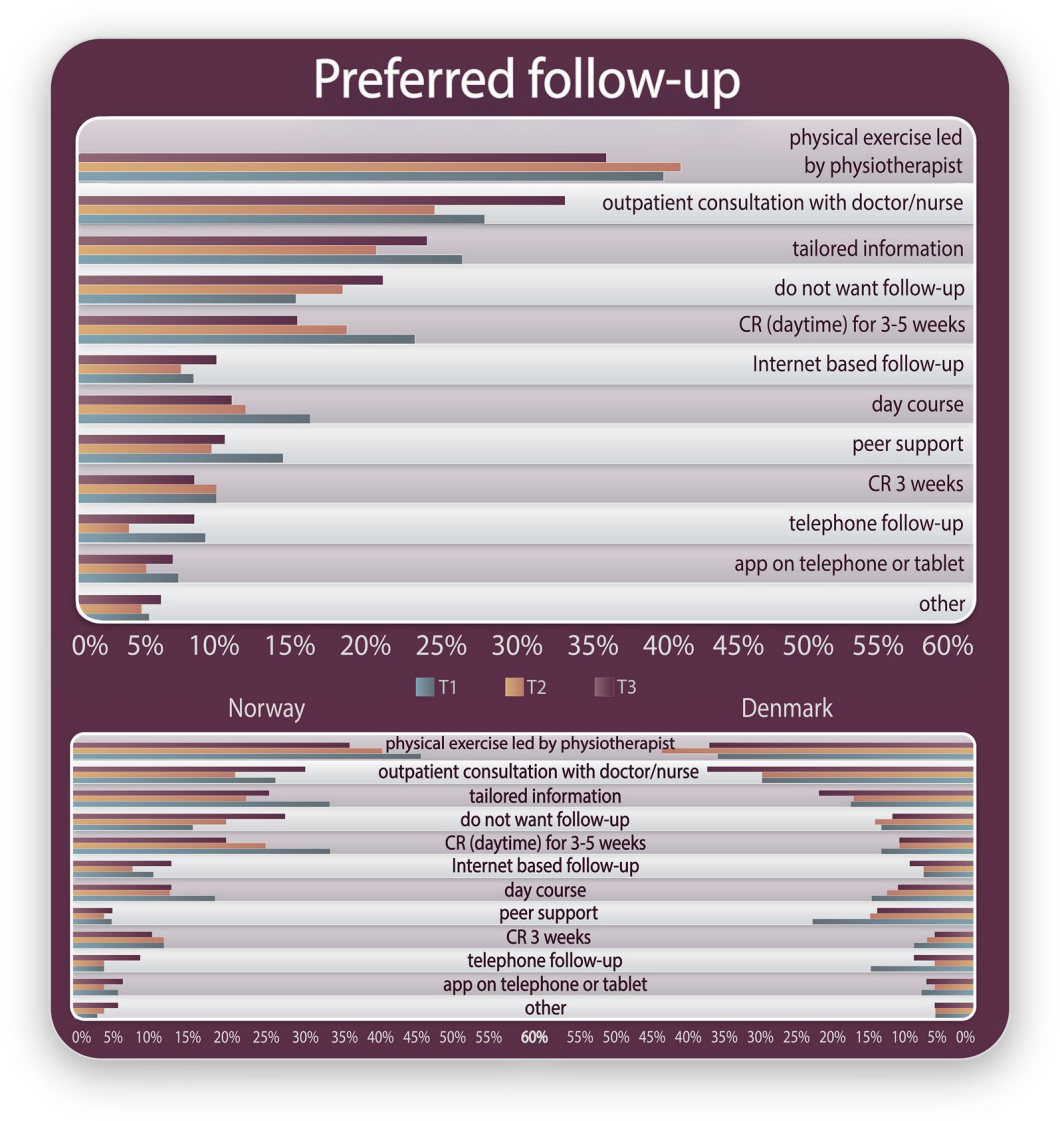
Type of follow-up patients reported that they prefer after percutaneous coronary intervention

Figure 2 shows the type of follow-up patients reported that they preferred (see Additional file 5 for Preferred follow-up in a table version). The most preferred follow-up was physical activity led by a physiotherapist at 2-, 6- and 12-month follow-up in both countries. At 2-month follow-up, *outpatient consultation, tailored information, CR (daytime) for 3–5 weeks* and *day courses* were the most preferred follow-up. At 12 months, *do not want follow-up* was among the five most reported responses for both Norwegian and Danish patients. At 2-month follow-up, 14% did *not want follow-up*, which increased to 20% at 12-month follow-up.

**Fig. 3:**
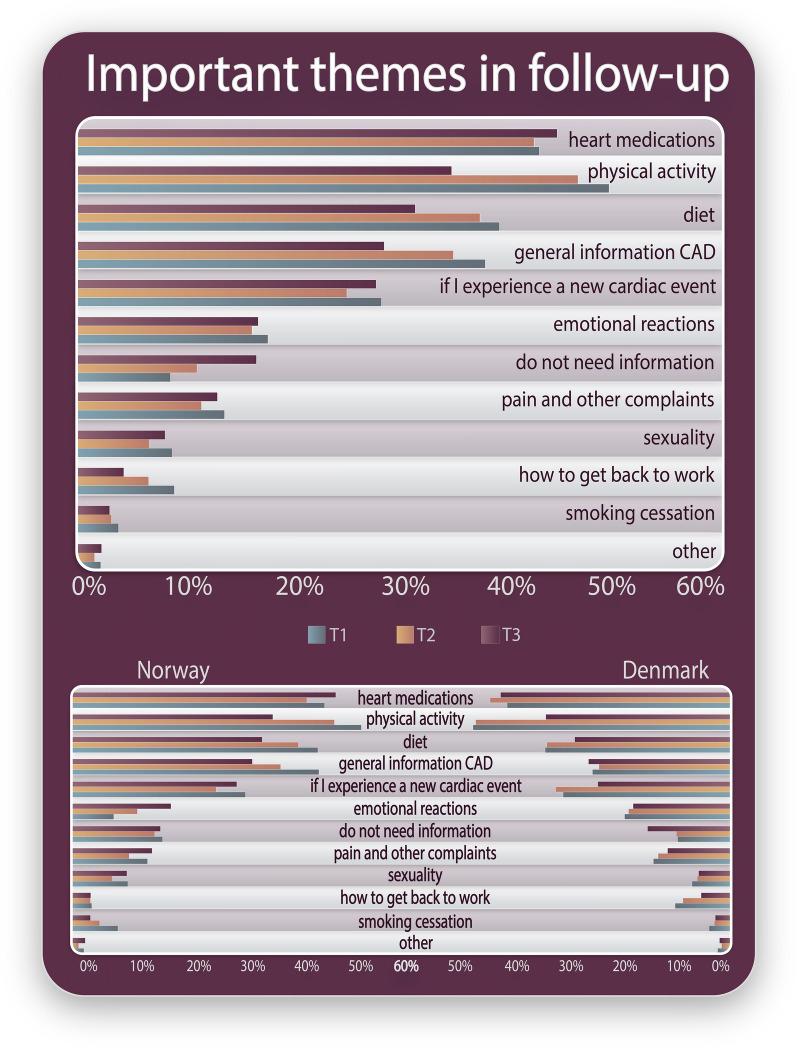
Themes patients reported to be important in follow-up after percutaneous coronary intervention

Figure 3 shows the themes patients considered important in follow-up, which were similar between the Norwegian and Danish patients at all measuring time points (see Additional file 6 for Important themes in follow-up in a table version). The top five themes in both countries at all measuring timepoints were *heart medications*, *physical activity*, *diet*, *general information about CAD*, and *what to do if they experienced a new cardiac event*.

From the index hospitalisation to 12-month follow-up, a lower proportion of patients reported that *physical activity*, *diet* and *general information about CAD* were important themes. Additionally, an increasing proportion reported that they *do not need information* over time.

## Discussion

**Fig. 4:**
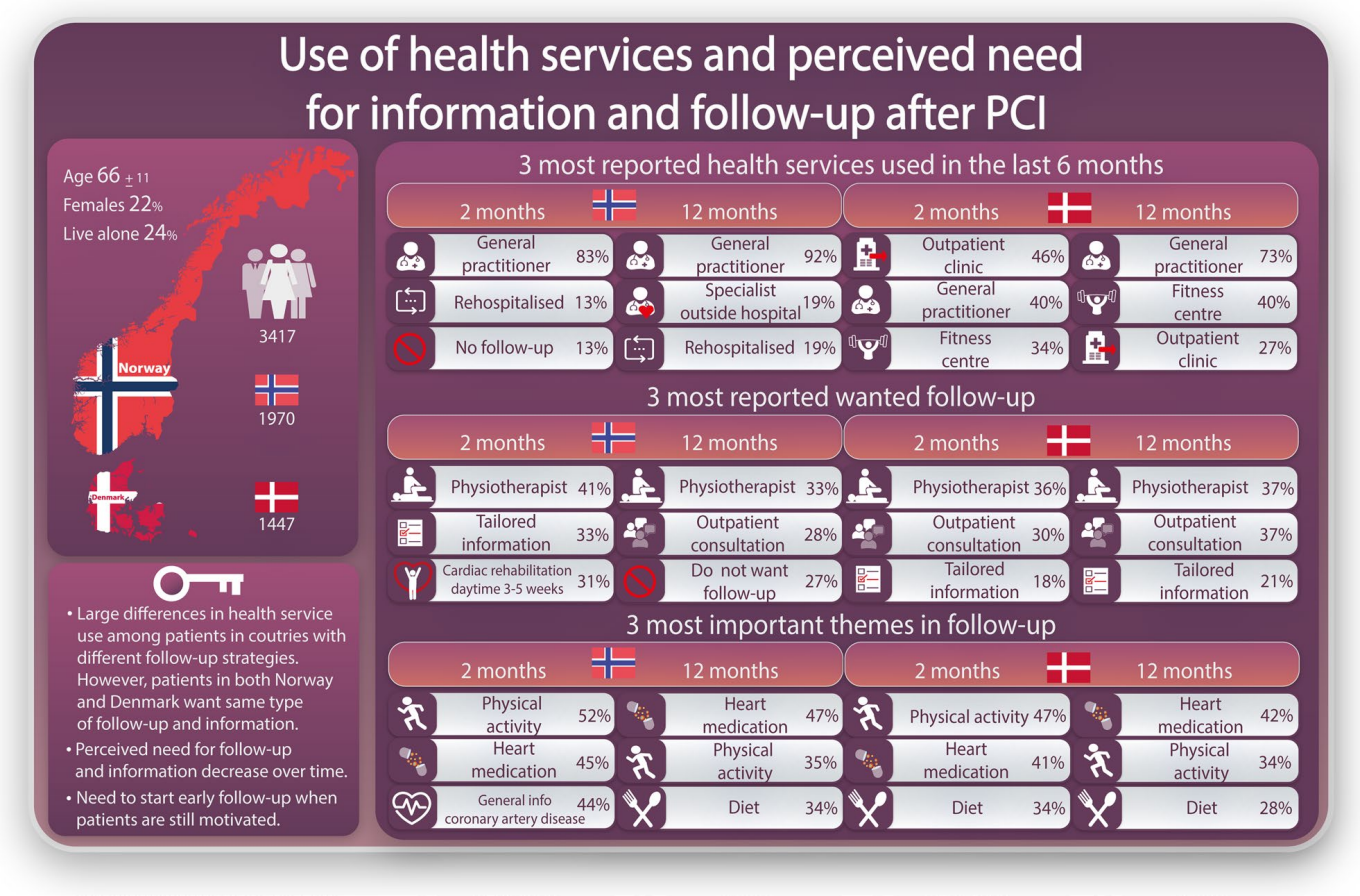
Summary of results

To our knowledge, few studies have investigated patients’ perceived need for follow-up and information after PCI. We found large differences in the type of health services patients had used in two countries with similar healthcare systems and reimbursement policies but with different follow-up practices after PCI. Norwegian patients to a larger extent visit their GP, while outpatient clinics are more commonly used in Denmark. Despite a more systematic referral of patients to CR, more patients in Denmark reported that they did not receive any follow-up. Although there are differences in the type of health services used, patients in Norway and Denmark preferred the same type of follow-up and valued the same type of information at 2-, 6- and 12-month follow-up, as shown in Fig. 4.

Patients’ hospital stay after PCI is short and they usually experience immediate relief from their symptoms. Thus, patients often think that they have been ‘fixed’[19]. The need for lifestyle changes may therefore not be that apparent. Several barriers to risk factor control and lifestyle changes have been identified, including psychosocial, clinical and accessibility issues [20, 21]. Healthcare providers’ beliefs about the benefit of CR might influence the information patients receive about CR and whether they are referred to CR or not [22, 23].

In some countries, including Norway, patients do not routinely participate in hospital-based CR or any other follow-up after PCI. Instead, they must contact their GP themselves. Lack of information flow between hospitals and patients, and hospitals and GPs impedes continuity of care [24]. This lack of systematically providing CR contributes to the notion that the patients’ condition is not that serious. In addition, during the waiting time for CR or other follow-up, patients’ motivation for lifestyle changes might have changed and they may already have slipped back into previous habits. Some patients might think that CR only corresponds to physical activity [25], making them reluctant to participate.

Our results show that physical activity led by a physiotherapist was the most preferred follow-up at all measuring time points in both countries. Physical activity is an important part of secondary prevention and CR strategies [2]. However, it should also be continued after structured follow-up has been completed.

Being aware of patients’ health literacy is also important when informing them about their chronic disease, as well as tailoring the information to the patient and their specific situation [26]. Through secondary prevention and CR, patients gain more knowledge about their condition, the importance of adhering to treatment and how to incorporate lifestyle changes. Tailoring the follow-up to older adults, females or those living far from a CR centre might increase referral, uptake and adherence. To ensure that patients are able to access, understand, appraise, remember and use the knowledge, they need to make informed choices regarding their own situation [27]. Thus, individualisation and longer-term follow-up than currently is provided, might be needed. The high proportion of patients who preferred follow-up on physical activity, diet and medications 12 months after PCI suggests that patients know that it is important but find it hard to adhere.

## Conclusion

There were large differences in the health services used in two countries with similar healthcare systems and reimbursement policies, but with different follow-up strategies after PCI. Patients in both countries found the same type of follow-up and information important. However, the perceived need for follow-up and information decreased over time.

This suggests a need to provide early structured follow-up when patients are still motivated. New modes of delivery and individual tailoring of secondary prevention strategies and CR are needed to overcome barriers to adherence. Primary and specialist healthcare providers should collaborate closely to implement secondary prevention strategies and CR after PCI and apply a long-term perspective to ensure that patients are able to self-manage their CAD risk profile and adhere to recommended treatment.

### Limitations

CONCARD^PCI^ is a large multicentre cohort study with broad inclusion criteria and 80% inclusion rate, as well as low attrition. However, the study is not without limitation. The retrospective nature of patients’ self-report might underreport use of health services. This study presents patients self-reported use of a range of health services, preferred health services and information important to them in follow-up care. However, these variables are presented descriptively without examining associations with sex, age or educational level, which is known to influence e.g., participation rates in CR. The study was conducted in two Nordic countries, which may limit its generalisability to countries without universal health coverage.

### Supplementary Information

**Additional file 1**. Flowchart CONCARD^PCI^ data collection.

**Additional file 2**. Comparison of participants and those declining participation in the study for the Norwegian centres.

**Additional file 3**. Patient characteristics.

**Additional file 4**. Patient reported use of health services after percutaneous coronary intervention.

**Additional file 5**. Type of follow-up patients reported that they prefer after percutaneous coronary intervention.

**Additional file 6**. Themes patients reported to be important in follow-up after percutaneous coronary intervention.

## Data Availability

The data that support the findings of this study are not openly available due to reasons of sensitivity to protect study participant privacy. Data are located in controlled access data storage at Haukeland University Hospital.
