# Supplementary figures and images for: Use of health services and perceived need for information and follow-up after percutaneous coronary intervention
Source: BMC Res Notes. 2024 Jan 5;17:20. doi: 10.1186/s13104-023-06662-y (PMC10768322; doi:10.1186/s13104-023-06662-y)

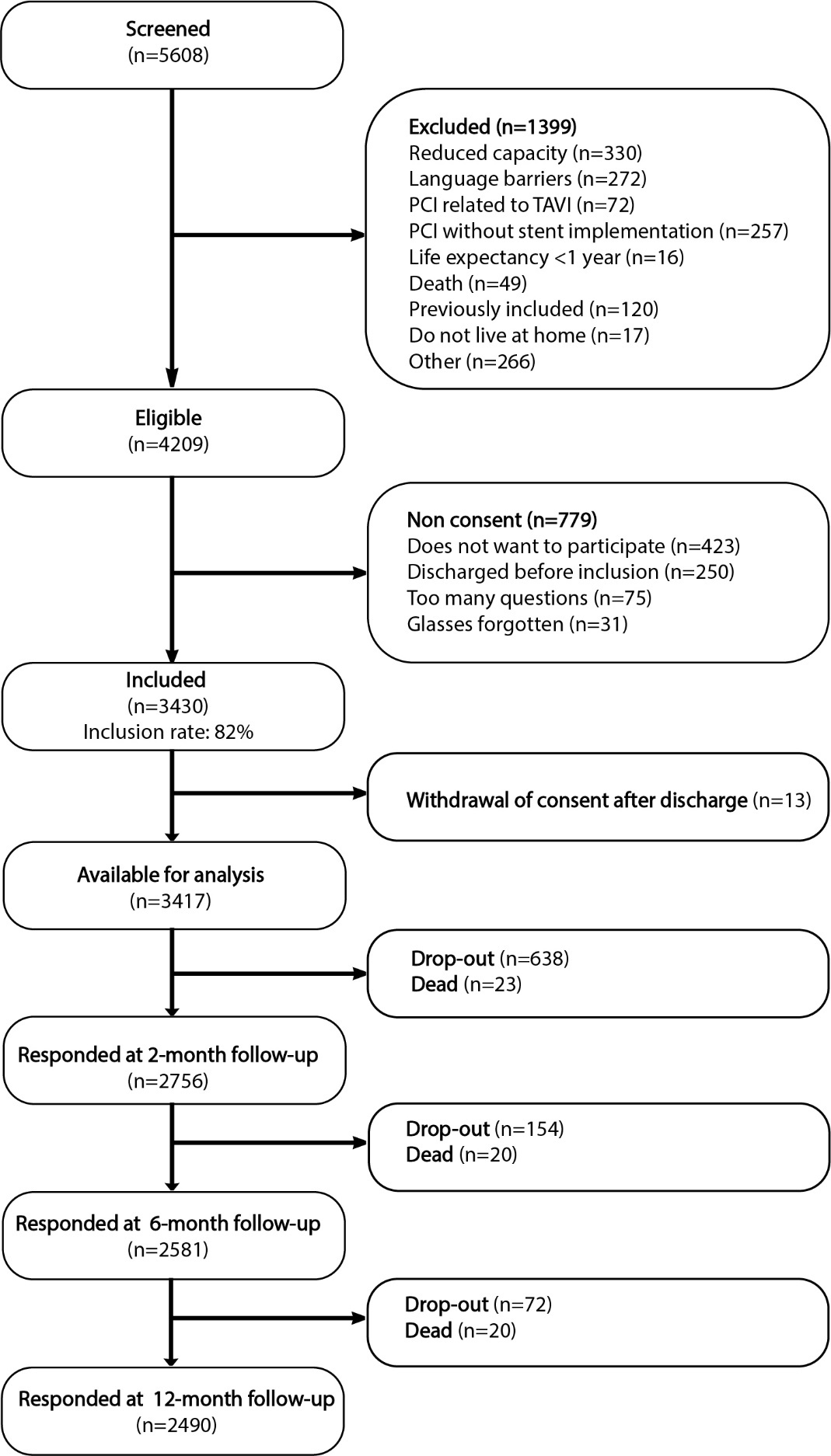


Supplementary 1 Flowchart CONCARDPCI data collection

Supplement: Supplementary file 1 — Additional file 1. Flowchart CONCARDPCI data collection. [file 13104_2023_6662_MOESM1_ESM.docx]
