# Supplementary material for: Use of health services and perceived need for information and follow-up after percutaneous coronary intervention
Source: BMC Res Notes. 2024 Jan 5;17:20. doi: 10.1186/s13104-023-06662-y (PMC10768322; doi:10.1186/s13104-023-06662-y)
Supplement: Supplementary file 2 — Additional file 2. Comparison of participants and those declining participation in the study for the Norwegian centres. [file 13104_2023_6662_MOESM2_ESM.docx]

**Supplementary 2. Comparison of participants and those declining participation in the study for the Norwegian centres.**

Logistic regression was performed to investigate relationships of sex, age and indication for percutaneous coronary intervention with participation.

^a^Joint adjustment for sex, age and indication.

^b^Odds ratio per 5 years.

^c^Other include arrhythmia examination, completion of previous percutaneous coronary intervention, heart failure/cardiomyopathy, non-specific chest pain, risk assessment following successful thrombolysis, suspected complications following angiography/percutaneous coronary intervention, valvular heart disease.

Abbreviations: CI: confidence intervals, OR: odds ratio, ref: reference, SD: standard deviation.

|  | **Participants (n=1970)**  **n (%)** | **Non-participants (n=582)**  **n (%)** | **OR** | **95% CI** | ***p*-value** | **OR** | **95% CI** | ***p*-value** |
| --- | --- | --- | --- | --- | --- | --- | --- | --- |
|  | **Descriptives** | | **Unadjusted analysis** | | | **Adjusted analysis^a^** | | |
| **Sex**  Female vs male (ref) | 1543 (78) | 428 (73) | 0.77 | 0.62 – 0.95 | 0.016 | 0.85 | 0.68 – 1.06 | 0.151 |
| **Age** (mean, SD)^b^ | 66 (11) | 69 (12) | 0.87 | 0.83 – 0.90 | <0.001 | 0.87 | 0.83 – 0.91 | <0.001 |
| **Indication for percutaneous coronary intervention**  Stable coronary artery disease (ref)  Unstable angina pectoris  Non-ST-segment elevation myocardial infarction  ST-segment elevation myocardial infarction  Other^c^ | 535 (27)  306 (16)  578 (29)  406 (21)  145 (7) | 120 (21)  86 (15)  183 (31)  105 (18)  84 (14) | 0.80  0.71  0.87  0.39 | 0.59 – 1.09  0.55 – 0.92  0.65 – 1.16  0.28 – 0.54 | <0.001    0.155  0.009  0.339  <0.001 | 0.79  0.74  0.79  0.37 | 0.57 – 1.08  0.57 – 0.96  0.59 – 1.07  0.27 – 0.52 | <0.001  0.133  0.022  0.127  <0.001 |
