# Supplementary material for: Use of health services and perceived need for information and follow-up after percutaneous coronary intervention
Source: BMC Res Notes. 2024 Jan 5;17:20. doi: 10.1186/s13104-023-06662-y (PMC10768322; doi:10.1186/s13104-023-06662-y)
Supplement: Supplementary file 3 — Additional file 3. Patient characteristics. [file 13104_2023_6662_MOESM3_ESM.docx]

**Supplementary 3. Patient characteristics**

| **Characteristics All** | | **Norway**  **N=1970** | **Denmark**  **N=1447** |
| --- | --- | --- | --- |
| **Sex**  Men | 2671 (78) | 1543 (78) | 1128 (78) |
| **Age, mean (SD)** | 66 ± 11 | 66 ± 11 | 66 ± 11 |
| **Living alone** | 750 (24) | 370 (21) | 380 (28) |
| **Education**  Primary school  Trade school  High school  University college or university, < 4 years  University college or university, ≥ 4 years | 640 (20)  1375 (43)  298 (9)  488 (15)  380 (12) | 369 (21)  660 (37)  187 (10)  307 (17)  265 (15) | 270 (19)  714 (51)  111 (8)  178 (13)  115 (8) |
| **Total household gross income (in NOK)**  < 125 000  125 000 - 200 000  201 000 - 300 000  301 000 - 400 000  401 000 - 550 000  551 000 - 700 000  701 000 - 850 000  >850 000 | 68 (2)  255 (8)  449 (15)  425 (14)  507 (17)  448 (15)  307 (10)  590 (19) | 19 (1)  52 (3)  181 (11)  226 (13)  309 (18)  277 (16)  202 (12)  438 (26) | 49 (4)  203 (15)  268 (20)  199 (15)  198 (15)  171 (13)  105 (8)  152 (11) |
| **Indication for PCI**  Stable coronary artery disease  Unstable angina pectoris  Non-ST-segment elevation myocardial infarction  ST-segment elevation myocardial infarction  Other | 1020 (30)  437 (13)  912 (27)  739 (22)  Other (9) | 535 (27)  306 (16)  578 (29)  406 (21)  145 (7) | 485 (34)  131 (9)  334 (23)  333 (23)  164 (11) |
| **Previous PCI** | 873 (26) | 488 (25) | 387 (27) |
| **Previous CABG** | 312 (9) | 196 (10) | 116 (8) |
| **Previous MI** | 704 (21) | 430 (22) | 274 (19) |
| **Years since first diagnosed with CAD, mean (SD)** | 3.48 ± 6.88 | 3.68 ± 7.25 | 3.19 ± 6.32 |
| **Body mass index, mean (SD)**  **BMI categories**  Underweight ( <18.5)  Healthy weight (18.5 – 24.9)  Overweight (25 – 29.9)  Obese class1 (30 – 34.9)  Obese class2 (35 – 39.9)  Obese class3 (≥40)  **Obese (BMI ≥ 30)** | 27.71 ± 4.50  22 (1)  937 (27)  1534 (45)  662 (19)  169 (5)  93 (3)  924 (27) | 27.49 ± 4.32  16 (1)  561 (28)  901 (46)  349 (18)  93 (5)  50 (3)  492 (25) | 28.02 ± 4.71  6 (0)  376 (26)  633 (44)  313 (22)  76 (5)  43 (3)  432 (30) |
| **Smoking status**  Current smoker  Former smoker  Never smoker | 529 (17)  1712 (54)  943 (30) | 273 (15)  978 (54)  553 (31) | 256 (19)  734 (53)  390 (28) |
|  |  |  |  |

Abbreviations: SD: standard deviationPCI: Percutaneous coronary intervention, CABG: coronary artery bypass graft, CAD: coronary artery disease, BMI: body mass index.
