# Supplementary material for: Use of health services and perceived need for information and follow-up after percutaneous coronary intervention
Source: BMC Res Notes. 2024 Jan 5;17:20. doi: 10.1186/s13104-023-06662-y (PMC10768322; doi:10.1186/s13104-023-06662-y)
Supplement: Supplementary file 4 — Additional file 4. Patient reported use of health services after percutaneous coronary intervention. [file 13104_2023_6662_MOESM4_ESM.docx]

**Supplementary 4. Patient reported use of health services after percutaneous coronary intervention.**

|  | **T1 (n=2717)** | **T2 (n=2566)** | **T3 (n=2448)** |
| --- | --- | --- | --- |
| **General practitioner** | **1747 (64)** | **1957 (76)** | **2054 (84)** |
| *Norway* | *1281 (83)* | *1344 (90)* | *1299 (92)* |
| *Denmark* | *466 (40)* | *613 (57)* | *755 (73)* |
| *p-value** | *p<0.001* | *p<0.001* | *p<0.001* |
| **Specialist outside the hospital** | **178 (7)** | **338 (13)** | **416 (17)** |
| *Norway* | *132 (9)* | *252 (17)* | *267 (19)* |
| *Denmark* | *46 (4)* | *86 (8)* | *149 (14)* |
| *p-value** | *p<0.001* | *p<0.001* | *P=0.003* |
| **Rehospitalised** | **359 (13)** | **415 (16)** | **441 (18)** |
| *Norway* | *197 (13)* | *253 (17)* | *264 (19)* |
| *Denmark* | *162 (14)* | *162 (15)* | *177 (17)* |
| *p-value** | *p=0.419* | *p=0.210* | *p=0.276* |
| **Psychologist/psychiatrist outside the hospital** | **43 (2)** | **91 (4)** | **66 (3)** |
| *Norway* | *19 (1)* | *48 (3)* | *27 (2)* |
| *Denmark* | *24 (2)* | *43 (4)* | *39 (4)* |
| *p-value** | *p=0.092* | *p=0.284* | p=0.006 |
| **Physical therapist outside the hospital** | **210 (8)** | **246 (10)** | **219 (9)** |
| *Norway* | *117 (8)* | *160 (11)* | *113 (8)* |
| *Denmark* | *93 (8)* | *86 (8)* | *106 (10)* |
| *p-value** | *p=0.746* | *p=0.022* | *p=0.062* |
| **Fitness center outside the hospital** | **595 (22)** | **720 (28)** | **582 (24)** |
| *Norway* | *192 (12)* | *280 (19)* | *169 (12)* |
| *Denmark* | *403 (34)* | *440 (41)* | *413 (40)* |
| *p-value** | *p<0.001* | *p<0.001* | *p<0.001* |
| **Private hospital** | **17 (1)** | **37 (1)** | **53 (2)** |
| *Norway* | *13 (1)* | *20 (1)* | *28 (2)* |
| *Denmark* | *4 (0)* | *17 (2)* | *25 (2)* |
| *p-value** | *p=0.101* | *p=608* | *p=0.481* |
| **Alternative treatment** | **24 (1)** | **27 (1)** | **49 (2)** |
| *Norway* | *6 (0)* | *11 (1)* | *20 (1)* |
| *Denmark* | *18 (2)* | *16 (2)* | *29 (3)* |
| *p-value** | *p=0.002* | *p=0.065* | *p=0.017* |
| **Community nursing** | **50 (2)** | **55 (2)** | **51 (2)** |
| *Norway* | *27 (2)* | *31 (2)* | *28 (2)* |
| *Denmark* | *23 (2)* | *24 (2)* | *23 (2)* |
| *p-value** | *p=0.685* | *p=0.782* | *p=0.700* |
| **Rekonvalisenssenter** | **11 (0)** | **16 (1)** | **9 (0)** |
| *Norway* | *10 (1)* | *13 (1)* | *7 (1)* |
| *Denmark* | *1 (0)* | *3 (0)* | *2 (0)* |
| *p-value** | *p=0.022* | *p=0.061* | *p=0.219* |
| **Nursing home (short-term)** | **9 (0)** | **11 (0)** | **8 (0)** |
| *Norway* | *9 (1)* | *11 (1)* | *7 (1)* |
| *Denmark* | *0* | *0* | *0* |
| *p-value** | *p=0.009* | *p=0.005* | *p=0.085* |
| **Outpatient clinic in the hospital** | **698 (26)** | **609 (24)** | **488 (20)** |
| *Norway* | *156 (10)* | *231 (15)* | *209 (15)* |
| *Denmark* | *542 (46)* | *378 (35)* | *279 (27)* |
| *p-value** | *p<0.001* | *p<0.001* | *p<0.001* |
| **None of the options** | **410 (15)** | **226 (9)** | **203 (8)** |
| *Norway* | *197 (13)* | *69 (5)* | *90 (6)* |
| *Denmark* | *213 (18)* | *157 (15)* | *113 (11)* |
| *p-value** | *p<0.001* | *p<0.001* | *p<0.001* |

* p-value from chi-square test.
