# Supplementary material for: Use of health services and perceived need for information and follow-up after percutaneous coronary intervention
Source: BMC Res Notes. 2024 Jan 5;17:20. doi: 10.1186/s13104-023-06662-y (PMC10768322; doi:10.1186/s13104-023-06662-y)
Supplement: Supplementary file 5 — Additional file 5. Type of follow-up patients reported that they prefer after percutaneous coronary intervention. [file 13104_2023_6662_MOESM5_ESM.docx]

**Supplementary 5. Type of follow-up patients reported that they prefer after percutaneous coronary intervention.**

|  | **T1 (N=2549)** | **T2 (N=2449)** | **T3 (N=2356)** |
| --- | --- | --- | --- |
| **Do not want follow-up** | **368 (14)** | **387 (18)** | **476 (20)** |
| *Norway* | 213 (15) | 274 (19) | 352 (27) |
| *Denmark* | 155 (13) | 113 (14) | 124 (12) |
| *p-value** | *p=0.105* | *p=0.005* | *p<0.001* |
| **Tailored information** | **623 (25)** | **493 (20)** | **493 (23)** |
| *Norway* | 425 (33) | 316 (22) | 273 (25) |
| *Denmark* | 198 (17) | 177 (17) | 220 (21) |
| *p-value** | *p<0.001* | *p<0.001* | *p=0.036* |
| **Day course** | **380 (16)** | **289 (12)** | **235 (11)** |
| *Norway* | 215 (17) | 162 (11) | 127 (12) |
| *Denmark* | 165 (14) | 127 (12) | 108 (10) |
| *p-value** | *p=0.053* | *p=0.737* | *p=0.363* |
| **CR (daytime) for 3-5 weeks** | **547 (22)** | **444 (18)** | **310 (15)** |
| *Norway* | 389 (31) | 325 (23) | 199 (18) |
| *Denmark* | 158 (13) | 119 (11) | 111 (11) |
| *p-value** | *p<0.001* | *p<0.001* | *p<0.001* |
| **CR 3 weeks** | **251 (10)** | **241 (10)** | **167 (8)** |
| *Norway* | 157 (12) | 177 (12) | 112 (10) |
| *Denmark* | 94 (8) | 64 (6) | 55 (5) |
| *p-value** | *p<0.001* | *p<0.001* | *p<0.001* |
| **Internet based follow up** | **199 (8)** | **173 (7)** | **217 (10)** |
| *Norway* | 122 (10) | 101 (7) | 128 (12) |
| *Denmark* | 77 (7) | 72 (7) | 89 (9) |
| *p-value** | *p=0.006* | *p=0.699* | *p=0.016* |
| **Outpatient consultation with doctor/nurse** | **659 (27)** | **591 (24)** | **688 (32)** |
| *Norway* | 306 (24) | 274 (19) | 301 (28) |
| *Denmark* | 353 (30) | 317 (30) | 387 (37) |
| *p-value** | *p=0.001* | *p<0.001* | *p<0.001* |
| **Telephone follow up** | **230 (9)** | **107 (4)** | **169 (8)** |
| *Norway* | 57 (4) | 56 (4) | 83 (8) |
| *Denmark* | 173 (15) | 51 (5) | 86 (8) |
| *p-value** | *p<0.001* | *p=0.322* | *p=0.568* |
| **Physical exercise led by physiotherapist** | **948 (39)** | **999 (40)** | **745 (35)** |
| *Norway* | 526 (41) | 522 (37) | 359 (33) |
| *Denmark* | 422 (36) | 477 (44) | 386 (37) |
| *p-value** | *p=0.007* | *p<0.001* | *p=0.040* |
| **Peer support** | **334 (14)** | **220 (9)** | **207 (10)** |
| *Norway* | 61 (5) | 54 (4) | 59 (5) |
| *Denmark* | 273 (23) | 166 (15) | 148 (14) |
| *p-value** | *p<0.001* | *p<0.001* | *p<0.001* |
| **App on telephone or tablet** | **163 (7)** | **113 (5)** | **133 (6)** |
| *Norway* | 78 (6) | 58 (4) | 74 (7) |
| *Denmark* | 85 (7) | 55 (5) | 59(6) |
| *p-value** | *p=0.268* | *p=0.214* | *p=0.290* |
| **Other** | **93 (4)** | **104 (4)** | **116 (5)** |
| *Norway* | 33 (3) | 51 (4) | 62 (6) |
| *Denmark* | 60 (5) | 53 (5) | 54 (5) |
| *p-value** | *p=0.001* | *p=0.095* | *p=0.618* |

* p-value from chi-square test.
