# Supplementary material for: Use of health services and perceived need for information and follow-up after percutaneous coronary intervention
Source: BMC Res Notes. 2024 Jan 5;17:20. doi: 10.1186/s13104-023-06662-y (PMC10768322; doi:10.1186/s13104-023-06662-y)
Supplement: Supplementary file 6 — Additional file 6. Themes patients reported to be important in follow-up after percutaneous coronary intervention. [file 13104_2023_6662_MOESM6_ESM.docx]

**Supplementary 6. Themes patients reported to be important in follow-up after percutaneous coronary intervention.**

|  | **T1 (N=2580)** | **T2 (N=2509)** | **T3 (N=2412)** |
| --- | --- | --- | --- |
| **Do not need information** | **220 (9)** | **276 (11)** | **398 (17)** |
| Norway | 108 (8) | 171 (12) | 243 (18) |
| Denmark | 112 (10) | 105 (10) | 155 (15) |
| *p-value** | *p=0.090* | *p=0.093* | *p=0.069* |
| **General information CHD** | **980 (38)** | **876 (35)** | **640 (28)** |
| Norway | 622 (44) | 533 (37) | 389 (32) |
| Denmark | 358 (31) | 343 (32) | 251 (24) |
| *p-value** | *p<0.001* | *p=0.007* | *p<0.001* |
| **Diet** | **1014 (39)** | **939 (37)** | **704 (31)** |
| Norway | 619 (44) | 580 (40) | 410 (34) |
| Denmark | 395 (34) | 359 (33) | 294 (28) |
| *p-value** | *p<0.001* | *p<0.001* | *p=0.005* |
| **Physical activity** | **1281 (49)** | **1168 (47)** | **780 (35)** |
| Norway | 732 (52) | 668 (47) | 429 (35) |
| Denmark | 549 (47) | 500 (47) | 351 (34) |
| *p-value** | *p=0.013* | *p=0.968* | *p=0.398* |
| **Sexuality** | **224 (9)** | **165 (7)** | **178 (8)** |
| Norway | 142 (10) | 103 (7) | 119 (10) |
| Denmark | 82 (7) | 62 (6) | 59 (6) |
| *p-value** | *p=0.006* | *p=0.163* | *p<0.001* |
| **Smoking cessation** | **95 (4)** | **78 (3)** | **67 (3)** |
| Norway | 51 (4) | 47 (3) | 41 (3) |
| Denmark | 44 (4) | 31 (3) | 26 (3) |
| *p-value** | *p=0.841* | *p=0.584* | *p=0.219* |
| **Heart medications** | **1113 (43)** | **1067 (43)** | **1002 (45)** |
| Norway | 634 (45) | 597 (42) | 566 (47) |
| Denmark | 479 (41) | 470 (44) | 436 (42) |
| *p-value** | *p=0.044* | *p=0.264* | *p=0.023* |
| **How to get back to work** | **233 (9)** | **163 (7)** | **95 (4)** |
| Norway | 116 (8) | 72 (5) | 40 (3) |
| Denmark | 117 (10) | 91 (8) | 55 (5) |
| *p-value** | *p=0.115* | *p<0.001* | *p=0.019* |
| **Pain and other complaints** | **352 (14)** | **288 (12)** | **289 (13)** |
| Norway | 189 (13) | 146 (10) | 172 (14) |
| Denmark | 163 (14) | 142 (13) | 117 (11) |
| *p-value** | *p=0.685* | *p=0.017* | *p=0.038* |
| **Emotional reactions** | **455 (18)** | **407 (16)** | **374 (17)** |
| Norway | 228 (16) | 209 (15) | 191 (16) |
| Denmark | 227 (19) | 198 (18) | 183 (18) |
| *p-value** | *p=0.031* | *p=0.009* | *p=0.242* |
| **What to do if I experience a new cardiac event** | **733 (28)** | **627 (25)** | **625 (28)** |
| Norway | 438 (31) | 370 (26) | 356 (29) |
| Denmark | 295 (25) | 257 (24) | 269 (26) |
| *p-value** | *p=0.001* | *p=0.299* | *p=0.064* |
| **Other** | **56 (2)** | **37 (1)** | **48 (2)** |
| Norway | 31 (2) | 20 (1) | 29 (2) |
| Denmark | 25 (2) | 17 (2) | 19 (2) |
| *p-value** | *p=0.920* | *p=0.694* | *p=0.348* |

* p-value from chi-square test.
